# Exploring the *MEN1* dependent modulation of caspase 8 and caspase 3 in human pancreatic and murine embryo fibroblast cells

**DOI:** 10.1007/s10495-021-01700-1

**Published:** 2021-12-08

**Authors:** Nele Wagener, Malte Buchholz, Philippe Bertolino, Chang X. Zhang, Pietro Di Fazio

**Affiliations:** 1 Department of Visceral Thoracic and Vascular Surgery, Philipps University Marburg, Baldingerstrasse, 35043 Marburg, Germany; 2 Department of Trauma Surgery, Orthopaedics and Plastic Surgery, University Medical Center Goettingen, Robert-Koch-Str. 40, 37099 Göttingen, Germany; 3 Department of Gastroenterology, Endocrinology and Metabolism, Philipps-University Marburg, 35043 Marburg, Germany; 4 Cancer Research Center of Lyon CRCL, French Institute of Health and Medical Research, 69008 Lyon, France

**Keywords:** MEN1, Caspases, Cell death, Spheroids, Pancreatic neuroendocrine neoplasia

## Abstract

*MEN1* mutation causes pancreatic neuroendocrine neoplasia and benign malignancies of the parathyroid, the adrenal cortex and pituitary gland. The transcriptional activity of its product menin promotes the expression of genes deputed to several cellular mechanism including cell death. Here, we focused on its implication in the activation of the initiator and executioner caspases after staurosporine mediated cell death in 2D and 3D human and murine cell models. The administration of staurosporine, a well-known inducer of apoptotic cell death, caused a significant reduction of BON1, QGP1 and HPSC2.2 cell viability. The transient knockdown of *MEN1,* performed by using a specific siRNA, caused a significant down-regulation of *CDKN1A* and *TP53* transcripts. The treatment with 1 µM of staurosporine caused also a significant down-regulation of *MEN1* and was able to restore the basal expression of *TP53* only in QGP1 cells. Transient or permanent *MEN1* inactivation caused a decrease of caspase 8 activity in BON1, HPSC2.2 cells and *MEN1*^*−/−*^ MEFs treated with staurosporine. Caspase 3/7 activity was suppressed after administration of staurosporine in *MEN1* knocked down HPSC2.2 and *MEN1*^*−/−*^ MEFs as well. The cleaved caspase 8 and caspase 3 decreased in human cells after *MEN1* knockdown and in *MEN1*^*−/−*^ MEFs. The treatment with staurosporine caused a reduction of the size of *MEN1*^+*/*+^ MEFs spheroids. Instead, *MEN1*^*−/−*^ MEFs spheroids did not show any significant reduction of their size. In conclusion, *MEN1* controls the activity of the initiator caspase 8 and the executioner caspase 3 in human and murine cells. Restoring of a functional *MEN1* and interfering with the apoptotic mechanism could represent a future strategy for the treatment of *MEN1*-related malignancies.

**Supplementary Information:**

The online version contains supplementary material available at 10.1007/s10495-021-01700-1.

## Introduction

Multiple endocrine neoplasia type 1 (MEN1) is a rare inherited tumour syndrome, causing different types of neuroendocrine malignancies in the pituitary gland, the pancreas, and the parathyroid glands [1, 2]. *MEN1* has been discovered the mostly frequent mutated gene in pancreatic neuroendocrine tumors [3, 4] and almost all of them are family related [5]. It localizes to chromosome 11q13 and consists of 10 exons encoding a 610-amino-acid protein called menin. Menin is ubiquitously expressed and is predominantly located in the nucleus in non-dividing cells [6]. It shows no homology with other known proteins and the mechanism by which its loss of function leads to MEN1 is still unclear. Menin interacts as a key scaffold protein cross talking with different transcription genes and interplaying with multiple different signal pathways [7]. Menin interacts also with transcription factors, such as activating protein-1 (AP-1), JunD, nuclear factor-κB (NF-κB), β-catenin, mothers against decapentaplegic (SMAD) family members, and estrogen receptor α (ERα) [8]. Furthermore, mTOR inhibitors, somatostatin analogues, tyrosine kinase inhibitors and epigenetic drugs have shown a significant efficacy in several model of MEN1-related pancreatic neuroendocrine neoplasia [9].

MEN1 has shown to be responsible for the encoding of caspase 8 gene, and mice expressing a monoallelic menin express a lower level of caspase 8 than the homozygous [10]. Despite some first evidence of the possible implication of *MEN1* in cell death mechanisms, the exact role of menin in apoptotic and alternative apoptotic mechanisms e.g. endoplasmic reticulum (ER) stress-mediated and autophagic cell death has not been described yet. Therefore, the aim of this study was to investigate the interaction between menin and apoptosis.

## Results

### Cytotoxic effects of staurosporine

**Fig. 1:**
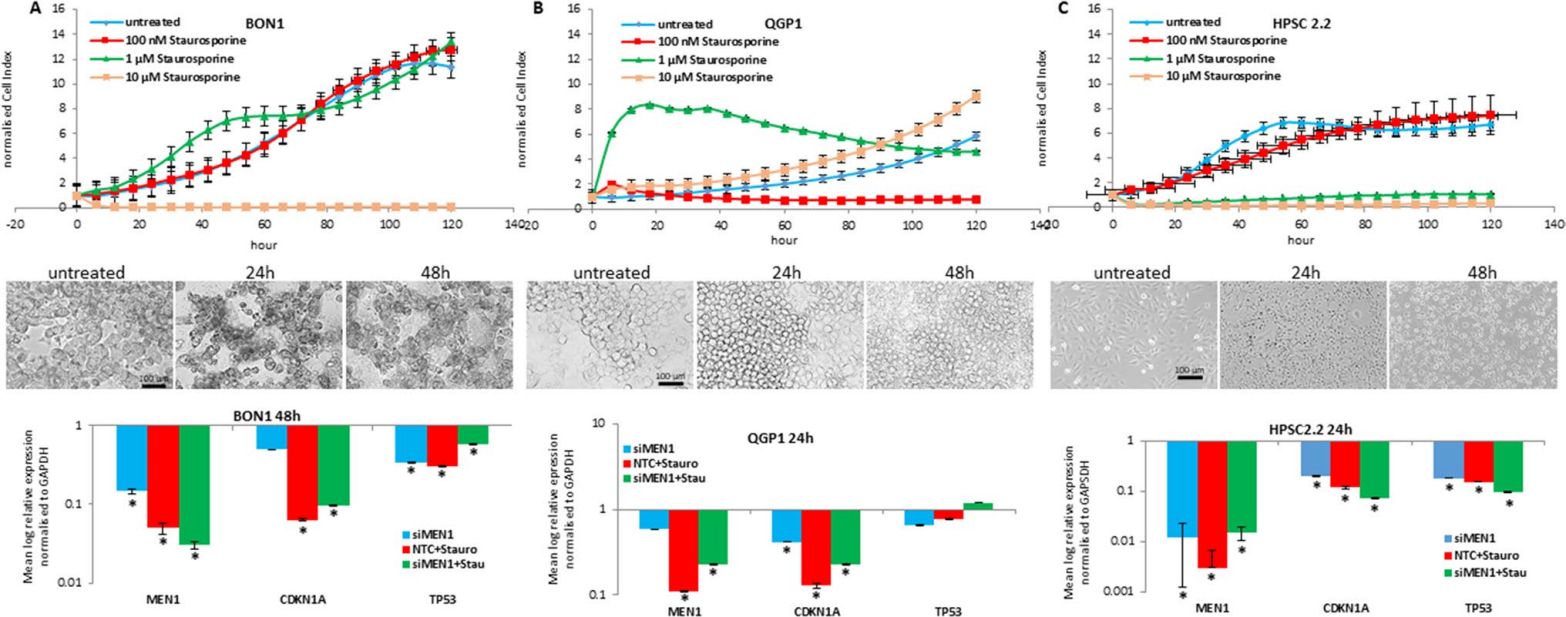
Effect of staurosporine on cell viability and transcripts expression. BON1 (**A**), QGP1 (**B**) and HPSC2.2 (**C**) cells were cultured in 96-well E-plates and were treated with 100 nM, 1 µM and 10 µM of staurosporine after 24 h. Cell impedance was measured continuously for 120 h. Shown are means of normalized cell index ± SD of three independent experiments performed in triplicates. BON1 (**A**), QGP1 (**B**) and HPSC2.2 (**C**) cells (middle panels) were treated with 1 µM of staurosporine for 24 h and 48 h. Inverted light microscopy pictures magnification is 100×. Scale bar represents 100 µm. Expression of *MEN1*, *CDKN1A* and *TP53* transcripts was determined in BON1 (**A**), QGP1 (**B**) and HPSC2.2 (**C**) monolayer cells treated for 24 h or 48 h with 1 µM of staurosporine and with a siRNA for *MEN1* (lower middle panel). Shown are means ± SEM of three independent experiments performed with biological duplicates. *p < 0.05 NTC (negative transfection control) vs siMEN1 or staurosporine treated cells

BON1, QGP1 and HPSC2.2 cell viability was continuously monitored for 120 h after administration of 100 nM, 1 µM and 10 µM of staurosporine. As shown in Fig. 1A, 10 µM of staurosporine was responsible for the significant reduction of the cell viability in BON1 cells. Instead, lower concentrations did not affect the cell viability, which showed a curve similar to untreated cells. The administration of 1 µM of staurosporine was monitored further by contrast light microscopy showing, in contrast to the cell viability, a reduction of cell attachment and density of BON1 cells after 24 h and 48 h of treatment. QGP1 cells (Fig. 1B) seemed not to be affected by 10 µM of staurosporine. Instead, the lowest concentration of 100 nM of staurosporine showed a strong reduction of cell viability. Interestingly, the light microscopy image showed a loss of QGP1 attachment after 24 h and 48 h of treatment with 1 µM of staurosporine.

Pancreatic stellate cells HPSC2.2 showed a reduction of cell viability after treatment with 1 and 10 µM of staurosporine. No reduction of cell growth was observed after treatment with 100 nM of staurosporine (Fig. 1C). Light microscopy images confirmed that 24 h and 48 h of treatment with 1 µM of staurosporine caused a detachment and a reduction of density of HPSC2.2 cells. The concentration of 1 μM of staurosporine was administered to the cells in all further experiments.

### Influence of *MEN1* and staurosporine on the expression of *TP53* and *CDKN1A*

An initial transfection with four specific siRNA for *MEN1* was performed to select the siRNA with the highest knockdown efficiency (Supplementary Figure). The most efficient siRNA, in terms of *MEN1* knockdown (Hs_MEN1_1) was used for the further experiments including the transient inactivation of *MEN1*. The transfection with the specific siRNA for *MEN1* caused a significant reduction (*p-value < 0.05) of *MEN1* and *TP53* transcripts in BON1 cells; HPSC2.2 cells showed a significant (*p-value < 0.05) down-regulation of *MEN1*, *CDKN1A* and *TP53* (Fig. 1A, C lowest panel). Treatment with 1 µM of staurosporine alone and after knock down of *MEN1* caused a significant (*p < 0.05) reduction of *MEN1*, *CDKN1A* and *TP53* in BON1 and HPSC2.2 cells. QGP1 cells showed a stable expression of *MEN1* transcript after transfection with a siRNA for *MEN1*. The treatment with 1 µM of staurosporine caused a significant reduction of *MEN1* and *CDKN1A*. *TP53* transcript level was stable.

### Expression of the caspases after treatment with staurosporine and *MEN1* knock down

**Fig. 2:**
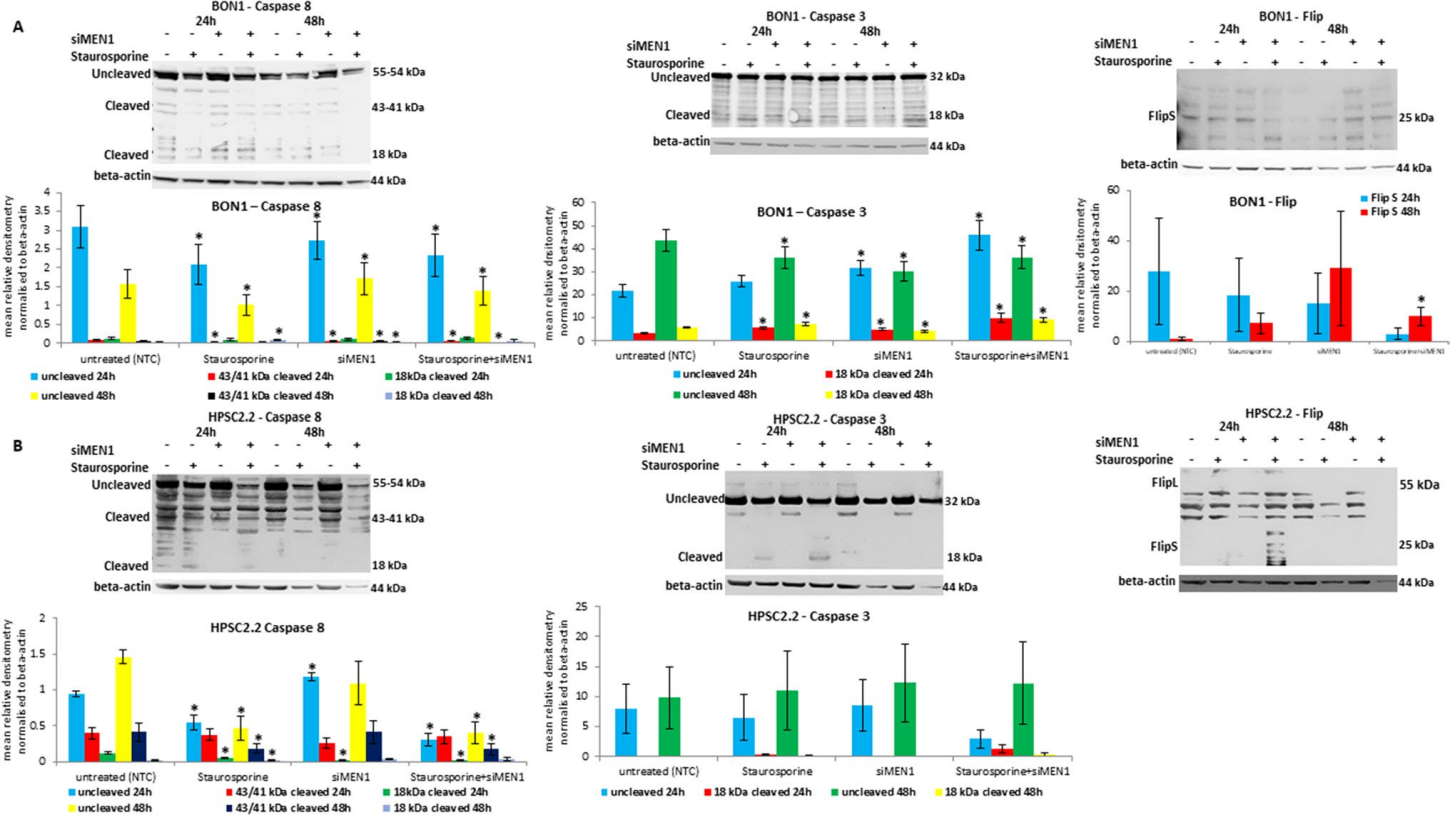
Staurosporine effect on caspases and Flip after *MEN1* knockdown. Expression and densitometry of caspase 8, caspase 3 and Flip in BON1 (**A**) and HPSC2.2 (**B**) cells treated for 24 h and 48 h with 10 µM of staurosporine and knocked down for *MEN1*. Densitometry results were normalized to β-actin content. *p < 0.05 NTC (negative transfection control) vs si*MEN1* or staurosporine treated cells

The protein level of caspase 8, caspase 3 and Flip was detected in BON1 and HPSC2.2 cells after *MEN1* knock down and exposure to 1 µM of staurosporine for 24 h and 48 h. As shown in Fig. 2A (left panels), the protein level of the uncleaved caspase 8 reduced significantly (p-value < 0.05) after 24 h of treatment with staurosporine, the knockdown of *MEN1* and their combined administration. The protein level of the 43/41 kDa cleaved caspase 8 was significantly down regulated by the treatment with 1 µM of staurosporine, the knock down of *MEN1* and their combination, whereas the protein level of the 18 kDa cleaved caspase 8 was unchanged. The longer exposure (48 h) to 1 µM of staurosporine caused a significant decrease of the uncleaved caspase 8. Instead, *MEN1* knock down caused a significant accumulation of it. The combined administration of staurosporine and si*MEN1* caused a significant decrease of the uncleaved caspase 8 in comparison to untreated cells. The 43/41 kDa cleaved caspase 8 strongly down regulated after exposure to staurosporine, *MEN1* knockdown and their combination. Interestingly, the 18 kDa cleaved caspase 8 was up regulated by the treatment with 1 µM of staurosporine. Instead, the knockdown of *MEN1* did not modulate its protein level that kept at the level of untreated cells, even after the addition of 1 µM of staurosporine. The protein level of the uncleaved caspase 3 (Fig. 2A middle panels) and its cleaved form was significantly up regulated after 24 h of exposure to 1 µM of staurosporine, *MEN1* knockdown and their combination. The longer exposure (48 h) to 1 µM of staurosporine, the *MEN1* knockdown and their combination caused a significant decrease of the uncleaved caspase 3. The protein level of the cleaved caspase 3 increased significantly after treatment with staurosporine. Instead, the knock down of *MEN1* caused a decrease of its protein level. The combined administration of 1 µM of staurosporine and si*MEN1* caused an up regulation of the cleaved caspase 3 as well.

The protein level of FlipL was not detectable in BON1 cells (Fig. 2A, right panels). The FlipS isoform decreased after 24 h of treatment with 1 µM of staurosporine, *MEN1* knockdown and their combination. The longer exposure (48 h) to staurosporine and siMEN1 caused an increase of its protein level even after both combined administration.

Human pancreatic stellate cells HPSC2.2 (Fig. 2B, left panels) showed a significant decrease of the uncleaved caspase 8 after 24 h of treatment with 1 µM of staurosporine alone and in combination with si*MEN1*. Instead, the solo knockdown of *MEN1* caused a significant increase of the uncleaved caspase 8 (Fig. 2B, left panels). The level of the 43/41 kDa cleaved caspase 8 was unchanged or slightly decreased after solo and combined administration of staurosporine and si*MEN1*. The protein level of the 18 kDa cleaved form was significantly (*p-value < 0.05) reduced by the treatment with staurosporine, si*MEN1* and their combined administration. The prolonged treatment with staurosporine (48 h) caused a significant (*p-value < 0.05) reduction of the uncleaved and both cleaved caspase 8 proteins even after knockdown of *MEN1*. Instead, the single incubation with siRNA for *MEN1* did not cause any change of the protein level of all forms of caspase 8. The protein level of the uncleaved caspase 3 was unchanged after 24 h of treatment with staurosporine or siMEN1 (Fig. 2B, middle panels). Their combination caused, instead, a reduction of its protein level. The longer exposure (48 h) caused no change of its protein level. The cleaved form of caspase 3 was modulated neither after 24 h nor after 48 h of treatment with 1 µM of staurosporine and/or siMEN1. Their combination caused only a slight increase of the cleaved caspase 3 after 24 h of treatment. FlipL was not detectable (Fig. 2B, right panel). The FlipS isoform was only detectable in HPSC2.2 cells treated for 24 h with 1 µM of staurosporine and si*MEN1*. The protein level of caspases and FlipS was densitometrically and statistically (t-test) analyzed and the results included as graphs included below the blots.

### Influence of *MEN1* on the caspases activity

**Fig. 3:**
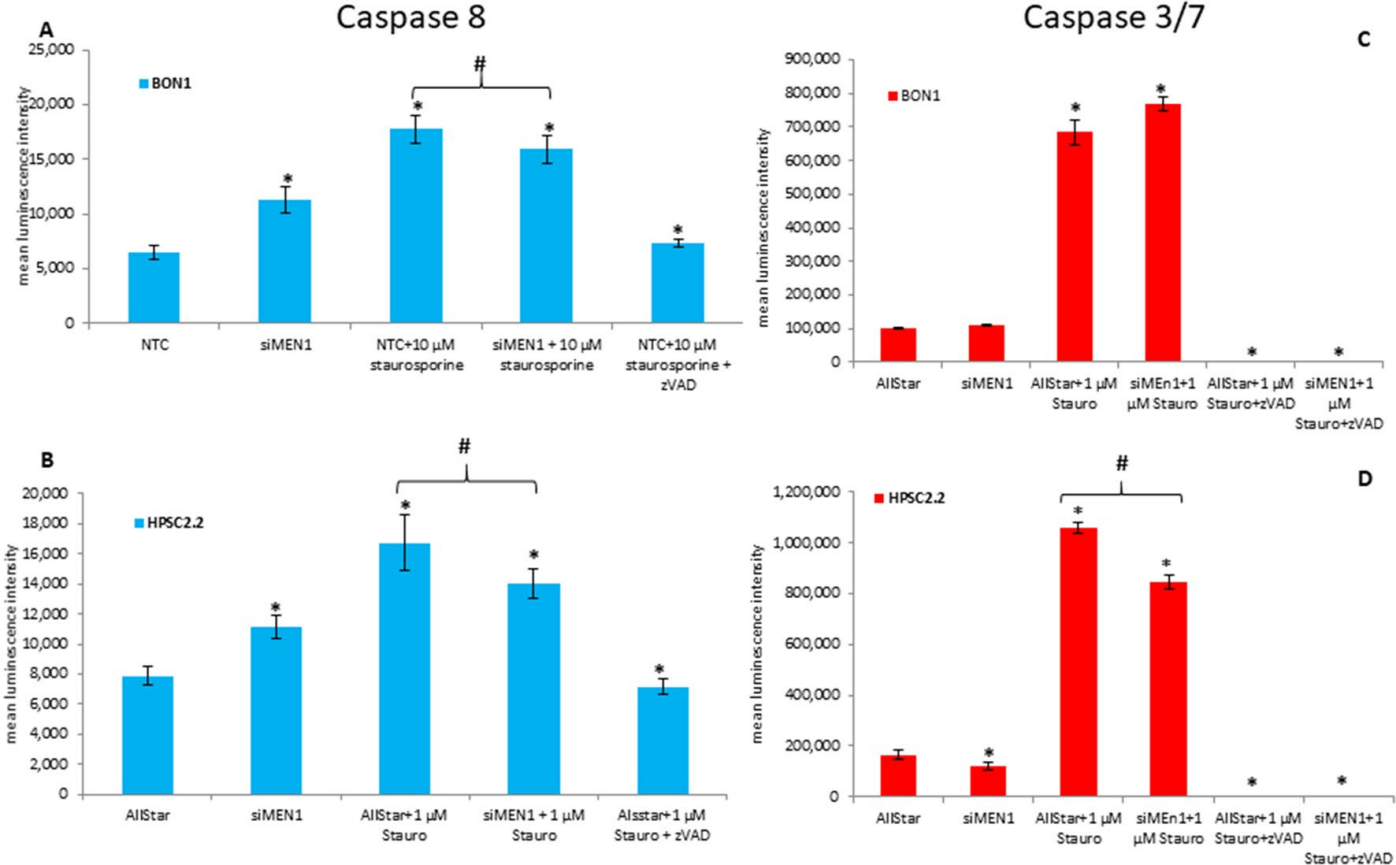
Caspases activity after *MEN1* knock down and staurosporine administration. Analysis of caspases 8 and 3/7 activity in BON1 (**A**, **C**) and HPSC2.2 (**B**, **D**) cells. ZVAD was included as negative control of caspase activity. *p < 0.05 NTC (negative transfection control) vs siMEN1 or staurosporine treated cells. #p < 0.05 staurosporine vs s*iMEN1* + staurosporine

BON1 and HPSC2.2 cells were transfected with a siRNA for *MEN1* and treated with 1 µM of staurosporine for up to 48 h. As shown in Fig. 3A and B, *MEN1* knock down or 24 h of treatment with 1 µM of staurosporine caused a significant (*p-value < 0.05) up-regulation of caspase 8 activity. Instead, the activity of caspase 8 was significantly (#p < 0.05) reduced by the combination of *MEN1* knockdown and staurosporine. ZVAD, a potent pan-caspase inhibitor, hampered the staurosporine-mediated activity of caspase 8. The activity of the executioner caspases 3 and 7 was further modulated by 48 h of treatment with 1 µM of staurosporine. BON1 cells showed an up-regulation of caspases 3/7 (Fig. 3C) after treatment with 1 µM of staurosporine. *MEN1* knockdown did not affect the efficacy of staurosporine. ZVAD reduced the caspase activity at lower level than the untreated sample. Instead, HPSC2.2 cells showed a significant (*p-value < 0.05) reduction of caspases 3/7 after *MEN1* knockdown (Fig. 3D). Furthermore, *MEN1* knockdown was able to block, significantly (#p < 0.05), the staurosporine-mediated hyper activation of the caspases. Once again, zVAD inhibited the activation of the caspases mediated by the treatment with 1 µM of staurosporine.

### Effect of staurosporine on MEF monolayer and spheroids

**Fig. 4:**
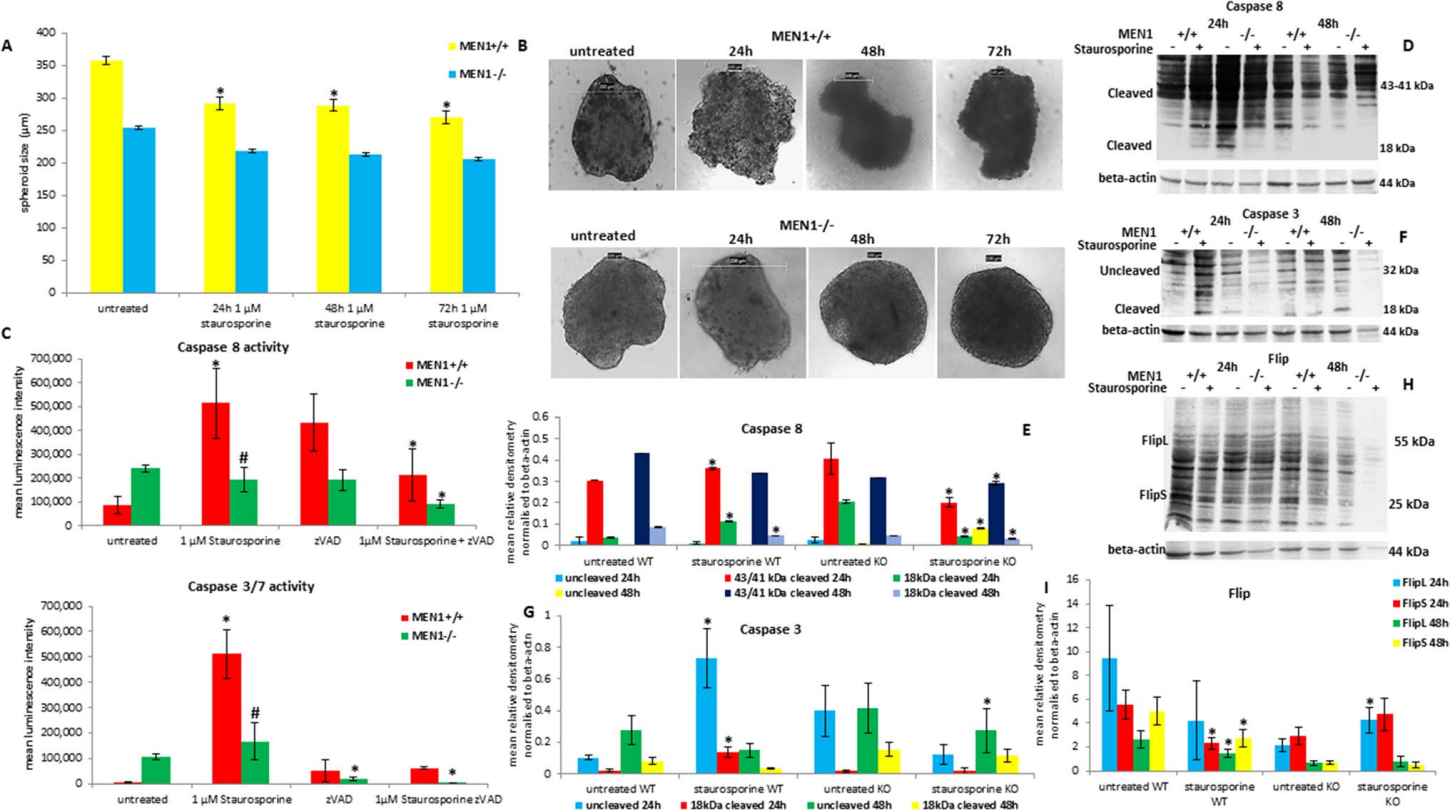
Effects of staurosporine in mouse embryo fibroblasts lacking *MEN1* expression. *MEN1*^+*/*+^ and *MEN1*^*−/−*^ mouse embryo fibroblasts spheroids size (**A**) was measured after 24 h, 48 h and 72 h of treatment with 1 µM of staurosporine. Shown are means ± S.D. of the size of 9 spheroids. *p < 0.05 untreated vs staurosporine treated spheroids. Light contrast microscopy of *MEN1*^+*/*+^ and *MEN1*^*−/−*^ MEF spheroids (**B**) after 24 h, 48 h and 72 h of treatment with 10 µM of staurosporine. Caspase 8 and caspase 3/7 activity (**C**) of *MEN1*^+*/*+^ and *MEN1*^*−/−*^ MEF monolayer treated for 48 h with 1 µM of staurosporine. Shown are means ± S.D. of experiments performed in triplicates. *p < 0.05 untreated vs staurosporine treated cells. Protein detection and densitometry of caspase 8 (**D**, **E**), caspase 3 (**F**, **G**) and Flip (**H**, **I**). Densitometry results were normalized to β-actin content. *p < 0.05 untreated vs staurosporine treated cells. #p < 0.05 *MEN1*^+*/*+^ vs *MEN1*^*−/−*^

Spheroids derived from *MEN1*^+*/*+^ and *MEN1*^*−/−*^ MEFs were treated for 72 h with 1 µM of staurosporine (Fig. 4A). The *MEN1*^+*/*+^ spheroids showed a significant (*p-value < 0.05) reduction of their size already after 24 h of treatment. The shrinking of the size was significant at all treatment time points. Instead, the *MEN1*^*−/−*^ spheroids showed no significant reduction of their size, even after 72 h of treatment with 1 µM of staurosporine. The Fig. 4B highlights that the treatment with 1 µM of staurosporine caused a reduction of the size, a loss of the integrity of the outer membrane and a dismantling of the ultrastructure of *MEN1*^+*/*+^ spheroids. Instead, *MEN1*^*−/−*^ spheroids were not affected by the treatment with 1 µM of staurosporine, they kept their ultrastructure and their outer membrane was intact. A small reduction of their size was the only detectable effect. *MEN1*^+*/*+^ and *MEN1*^*−/−*^ monolayer MEFs were tested for the caspases activity. As shown in Fig. 4C, 48 h of treatment with 1 µM of staurosporine caused a significant (*p < 0.05) increase of the caspases 8 and 3/7 in *MEN1*^+*/*+^ fibroblasts. Instead, *MEN1*^*−/−*^ cells showed no increase of the caspases activity after treatment with staurosporine, which was significantly (#p < 0.05) lower in comparison with *MEN1*^+*/*+^ cells. ZVAD was able to inhibit significantly (*p < 0.05) the activation of the caspases in the *MEN1*^+*/*+^ cells.

The uncleaved protein level of caspase 8 (Fig. 4D, E) slightly reduced in both MEFs after 24 h of treatment with staurosporine. The prolonged treatment (48 h) with 1 µM of staurosporine caused a significant (p-value < 0.05) increase of uncleaved caspase 8 protein level in *MEN1*^*−/−*^ cells. The 43/41 kDa cleaved caspase 8 increased significantly in *MEN1*^+*/*+^ cells, whereas decreased significantly in *MEN1*^*−/−*^ cells after 24 h of treatment with 1 µM of staurosporine. The longer exposure to staurosporine caused no significant change of its protein level in *MEN1*^+*/*+^ cells, and its level was significant (p-value < 0.05) stable in *MEN1*^*−/−*^ fibroblasts. The 18 kDa cleaved form of caspase 8 was characterized by a significant up-regulation in *MEN1*^+*/*+^ fibroblasts; instead, it decreased significantly in *MEN1*^*−/−*^ fibroblasts after 24 h of exposure to 1 µM of staurosporine. The 48 h treatment caused a significant decrease of the 18 kDa cleaved form in both *MEN1* wild type and knocked out fibroblasts. Caspase 3 showed a significant up-regulation of its uncleaved (32 kDa) and cleaved form (18 kDa) in *MEN1*^+*/*+^ cells after 24 h of treatment with 1 µM of staurosporine. The level of both proteins reduced after long time exposure (48 h). Interestingly, caspase 3 showed a down-regulation of both uncleaved and cleaved forms in *MEN1*^*−/−*^ fibroblasts after 24 h and 48 h of exposure to 1 µM of staurosporine (Fig. 4F, G). Both L and S isoforms of Flip were significantly down regulated in *MEN1*^+*/*+^ fibroblasts after 24 h and 48 h of treatment with 1 µM of staurosporine. *MEN1*^*−/−*^ fibroblasts showed, after 24 h of exposure, an increase of both isoforms, whereas no changes have been observed after 48 h of exposure to 1 µM of staurosporine (Fig. 4H, I). The protein level was densitometrically and statistically (t-test) analyzed and the results included as graphs included below the blots.

### Implication of *MEN1* in staurosporine-mediated apoptotic cell death

**Fig. 5:**
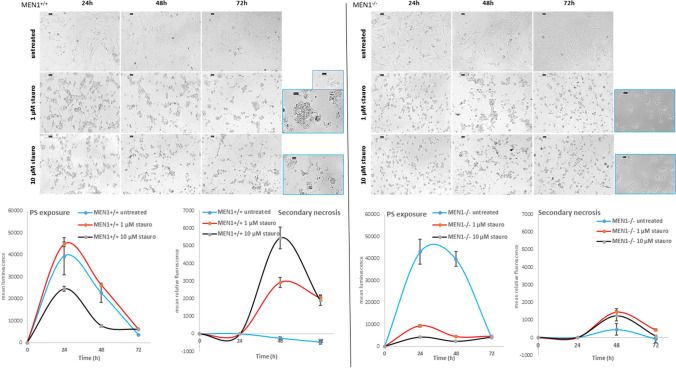
Detection of apoptosis in *MEN1*^+*/*+^ and *MEN1*^*−/−*^ MEFs. (Upper panels) Micrographs of *MEN1*^+*/*+^ and *MEN1*^*−/−*^ MEFs after the administration of 1 and 10 µM of staurosporine for up to 72 h. Higher magnification miniatures (blue frame) highlight the appearance of blebbing, apoptotic bodies and pyknosis in *MEN1*^+*/*+^ MEFs and their absence in *MEN1*^*−/−*^ MEFs. Scale bars are 50 and 20 µm. Magnification is 200× and 400×. (Lower panels) Detection of the luminescent signal of phosphatidylserine (PS) exposure and the fluorescent signal of secondary necrosis in *MEN1*^+*/*+^ and *MEN1*^*−/−*^ MEFs after the administration of 1 and 10 µM of staurosporine for up to 72 h. Shown are means of relative luminescence and fluorescence normalized to blank ± SEM of experiments performed in quadruplicates

The *MEN1*^+*/*+^ and *MEN1*^*−/−*^ MEFs were treated for up to 72 h with 1 and 10 µM of staurosporine. The micrographs (Fig. 5 upper panels) show that *MEN1*^+*/*+^ MEFs were strongly affected by the cytotoxicity of both concentrations of staurosporine already after 24 h of treatment. The prolonged treatment caused a further reduction of the cell number and the appearance of the typical apoptotic sub-cellular products, e.g. blebbing, apoptotic bodies and finally pyknosis. Instead, the *MEN1*^*−/−*^ MEFs were only characterized by a reduction of cell number. No apoptotic effects could be observed. Furthermore, the ongoing apoptosis could be proven in *MEN1*^+*/*+^ MEFs by the luminescence-based measurement of phosphatidylserine (PS) exposure and by the fluorescence-based (DNA-binding green fluorescent dye) measurement of secondary necrosis (Fig. 5 lower panels). These cells evidenced an increase of PS exposure after 24 h of treatment with both concentrations of staurosporine accompanied by an increase of the secondary necrosis after 48 h of treatment, thus confirming that the cells are dying by apoptosis. Instead, the untreated cells showed only an increase of PS, which was followed by an absent necrosis fluorescent signal. Interestingly, the *MEN1*^*−/−*^ MEFs evidenced a weak/absent increase of the PS exposure luminescent signal after the administration of both concentrations of staurosporine at all treatment time points, especially in comparison to the luminescent signal of untreated cells. Furthermore, the necrosis fluorescent signal detected in *MEN1*^*−/−*^ MEFs was weak in both untreated and staurosporine treated cells in comparison to *MEN1*^+*/*+^ MEFs (Fig. 5 lower panels).

These results highlight that the lack of *MEN1* confers the MEFs the resistance to the staurosporine cytotoxicity. These cells are able, after short time, to overcome the cytotoxicity caused by the treatment with staurosporine and in particular to survive to apoptosis.

## Discussion

Impairment of *MEN1* expression caused by mutation is responsible for the development of benign neoplasia of the parathyroid, pituitary and adrenocortical glands and further pancreatic neuroendocrine neoplastic lesions. *MEN1* mutation represents the most frequent alteration in pancreatic neuroendocrine malignancies [3]. It has been shown that *MEN1* is implicated in the regulation of factors involved in several cellular processes, e,g, cell proliferation, differentiation and death [3, 7]. However, its pro-death role has not been well defined yet. The first evidence of its implication in cell fate decision has been demonstrated in mouse embryo fibroblasts. The ectopic expression of menin, the protein encoded by *MEN1*, was responsible to mediate apoptosis by modulating the pro-apoptotic factors Bax and Bak [11]. Further evidence highlighted that point mutations occurring at *MEN1* impair its ability to promote the transcription of caspase 8 gene [12] and TNF-alpha-mediated apoptosis [10]. Furthermore, treatment with enalapril and aspirin increased the expression of caspase 3 in *MEN1*^+*/T*^ knock out mouse model [13]. The present study focused on the role exerted by *MEN1* under pro-apoptotic condition mediated by the treatment with staurosporine, a canonical apoptosis inducer in solid malignancies [13–16]. Transiently knockdown of *MEN1* caused a significant down-regulation of *CDKN1A* and *TP53* transcripts, as well as after treatment with staurosporine, proving a direct correlation between *MEN1* and the master regulators of cell fate as previously found in gamma-irradiated rat insulinoma cells where, *MEN1* mutated variants lowered the apoptotic response [17]. Additional findings evidenced that the treatment with menin-MLL inhibitor caused the restoration of proliferation mediated by suppression of *CDKN1A*, *CDKN1B* and *CDKN2C* in human pancreatic beta cells [18]. Furthermore, the knockdown of *MEN1* was responsible for the down-regulation of caspase 8 activity in human insulinoma cells and human pancreatic stellate cells. In addition, the stellate cells showed a down-regulation of the caspase 3 activity after *MEN1* knock down. These findings were further supported by the significant down-regulation of the protein level of the cleaved form of the caspases 8 and 3 in *MEN1* knocked down human cells treated with staurosporine. Additionally, the accumulation of the anti-apoptotic FlipS isoform confirmed that *MEN1* knockdown impedes cell demise, especially in human insulinoma cells. Thus introducing a new regulatory mechanism of MEN1 on the activity of caspases and expanding the previously described role as promoter of caspase 8 gene expression. Further results highlighted the role exerted by *MEN1* on the modulation of caspases. Interestingly, the knockout of *MEN1* in mouse embryo fibroblasts sustained these cells for being resistant to the pro-death treatment with staurosporine. As well, spheroids derived from *MEN1*^*−/−*^ fibroblasts were not perturbed by treatment with staurosporine, keeping an intact ultrastructure and a stable outer spheroid membrane. Furthermore, the loss of *MEN1* determined a down-regulation of both caspase 8 and caspase 3 activity and the protein level of their cleaved forms. Additionally, the two Flip isoforms L and S, exerting a fine tuned role in the modulation of apoptosis [19], were up regulated supporting that the loss of MEN1 inhibits the activation of pro-apoptotic players and let stabilize the anti-apoptotic markers. Further results evidenced that the lack of *MEN1* impeded, in MEFs, the exposure of phosphatidylserine and the secondary necrosis after the administration of staurosporine. Instead, the expression of *MEN1* determined, in staurosporine-treated MEFs, the accumulation of apoptotic bodies and the blebbing together with the increase of PS exposure and secondary necrosis.

In summary, this study shows that *MEN1* controls the activity of the initiator caspase 8 and the executioner caspase 3 in human and murine cells. Furthermore, the knockdown of *MEN1* caused a significant down-regulation of *CDKN1A* and *TP53* transcripts. Further studies need to confirm the relevance of these results in *MEN1* patients.

## Conclusion

The present study indicates that menin exerts an influence on the apoptotic-signaling pathway. Loss of menin blocks the apoptotic process. These new insights could highlight new therapeutic approaches in MEN1 patients.

### Methods and materials

#### Cell culture

Human insulinoma BON1, and pancreatic stellate cells HPSC2.2 were a kindly gift from Heidi Griesmann (Department of Gastroenterology, University Hospital Halle, Germany) and Malte Buchholz (Department of Gastroenterology, Philipps University Marburg Germany). The cells were grown in DMEM (Gibco, Paisley, UK) supplemented with 10% fetal bovine serum, penicillin (100 Units/ml) and streptomycin (100 µg/ml) at 37 °C in a humidified atmosphere containing 5% CO_2_. The human somatostatinoma cell line QGP1 (Malte Buchholz) were grown in RPMI Medium 1640 (Gibco® by Life Technologies™, Carlsbad, USA), with 10% fetal bovine serum, penicillin (100 Units/ml) and streptomycin (100 µg/ml). *MEN1*^+*/*+^ and *MEN1*^*−/−*^ mouse embryo fibroblasts, a kindly gift from Philippe Bertolino and Chang X. Zhang (Cancer Research Center of Lyon, France) [20], were grown in DMEM (Gibco) supplemented with 10% fetal bovine serum, penicillin (100 Units/ml) and streptomycin (100 µg/ml) at 37 °C in a humidified atmosphere containing 5% CO_2_.

### Substances

Staurosporine (SIGMA-ALDRICH, St Louis USA) and the Caspase Inhibitor zVAD-FMK (R&D Systems, Minneapolis USA) were dissolved in sterile DMSO (WAK-Chemie Medical GmbH, Steinbach Germany).

### Real-time cell viability analysis

BON1, QGP1 and HPSC2.2 cells were cultured on E-plates (05232368001, OLS, Bremen, Germany) and real-time cell viability was measured after treatment with 100 nM, 1 µM and 10 µM staurosporine by xCELLigence RTCA system (Roche, Basel Switzerland). XCELLigence continuously measured (120 h) the impedance to quantify the adherence of the cells on the plate´s electrodes.

### *MEN1* transient knockdown

Four specific siRNA targeting the transcript of *MEN1* (SI00630231, FlexiTube GeneSolution GS4221 for MEN1 Qiagen, Hilden, Germany) were used to perform the transient knockdown of MEN1 in human pancreatic cells BON1, QGP1 and pancreatic stellate cells HPSC2.2. The cells were transfected by following the fast transfection protocol suggested by the manufacturer (Qiagen). HiPerfect (Qiagen) was used as transfection reagent and mixed together with the specific siRNAs and serum free medium by following the manufacturer instrctions. The cells were first seeded with complete growth medium and then the transfection mixture was added. The knockdown was monitored by RT-qPCR after 48 h of transfection. The siRNA with the highest knockdown efficiency (Hs_MEN1_2, SI00630238, Qiagen) was used for the further experiments. A negative transfection control (NTC, Qiagen) was included in all experimental settings including the MEN1 knockdown.

### Quantitative RT-PCR

Total RNA was isolated with the RNeasy Mini Kit (74106, QIAGEN, Hilden Germany) according to the manufacturer`s protocol. Reverse Transcription of mRNA was performed with iScript™ cDNA Synthesis Kit (170-8891, Bio-Rad Laboratories, Hercules, USA) on FlexCycler (Analytik Jena AG, Jena, Deutschland). Qiagen primers for human *MEN1* (QT00064848), *CDKN1A* (QT00062090), *TP53* (QT00060235) and *GAPDH* (QT01192646) were used with GoTaq® qPCR Master Mix (Promega, Madison, USA) on RT-qPCR thermocycler CFX96™ Real-Time System (Bio-Rad Laboratories). Results were analysed with the Bio-Rad CFX-Manager (Bio-Rad Laboratories) and normalized with GAPDH mRNA content for each sample. Raw data were further analysed with Rest2009 (relative Expression Software Tool V.2.0.13. Qiagen).

### Western blot analysis

Whole cell lysates were prepared with Jie´s Buffer (10 mM NaCl, 0.5% NonidetP40, 20 mM Tris–HCL pH7.4, 5 mM MgCl_2_, 1 mM PMSF, Complete Protease Inhibitor and Phosphatase Inhibitor (Roche). The proteins were separated through SDS-Page (NP0342, Life Technologies, Carlsbad, California USA) and transferred to nitrocellulose membranes (10600009, GE Healthcare Life science, Chicago, USA) by semi-dry-blotting with Trans-Blot®Turbo™ Transfer System (Bio-Rad Laboratories). The membranes were further sliced according to the required molecular weight of the proteins of interest, blocked in 4% BSA (23208, Thermo Fisher Scientific, Waltham MA USA) in TBS-Tween20 (0.5%) and incubated with primary antibodies against Caspase 8 (ALX-804-242, Enzo Life Sciences GmbH, Lörrach Germany), Caspase 3 (NB100-56708, Novus Biologicals, Abingdon UK), Flip (ALX-804-961-0100, Enzo Life Sciences), β-actin (A5441 SIGMA-ALDRICH, St Louis USA) and GAPDH (ab9485, AbCam). Bound primary antibodies were detected by secondary horseradish-labelled goat anti-rabbit (A0545, SIGMA-ALDRICH), goat anti-mouse (A9917, SIGMA-ALDRICH) antibodies and SuperSignal West Pico Chemiluminiscent Substrate (Thermo Fisher Scientific, Waltham, USA). The immuno-detection was quantified using Fusion image capture (VILBER LOURMAT Deutschland GmbH, Eberhardzell, Germany) and Bio-1D analysis System (VILBER LORUMAT Deutschland GmbH).

### Caspase activity detection

The cells were seeded in 96-well cell culture plates and transfected with the siRNA for *MEN1*. Caspase activity was determined after treatment with staurosporine (6–48 h) with the Caspase Glo-8 (G8200) and the Caspase Glo-3/7 (G8090) assays from Promega by following the manufacturer instructions.

### Establishing of MEF-derived spheroids

*MEN1*^+*/*+^ and *MEN1*^*−/−*^ mouse embryo fibroblasts spheroids were formed on 50 µl 1.5% peqGOLD Universal Agarose (PEQLAB Biotechnology GmbH, Erlangen, Deutschland) in a flat-bottom 96-well plate (SARSTEDT AG and Co. KG, Nümbrecht, Germany) for 6 days without medium change [21]. MEFs were placed on an orbital shaker with a shaking speed of 40 rpm overnight. Before treatment, 100 µl of medium were removed from each well containing a single spheroid. 100 µl medium with staurosporine were added to the remaining 100 µl medium. The working concentration was 10 µM staurosporine. The micrographs were acquired by inverted microscope Leica (Wetzlar, Germany) with a Leica EC3 camera and digitalized by the software LAS EZ version 2.1.0.

### Measurement of apoptosis/necrosis

The detection of apoptosis/necrosis was performed by luminescence/fluorescence after the administration of the RealTime-Glo™ Annexin V Apoptosis and Necrosis Assay (JA1011, Promega). 10,000 MEFs (MEN1 + / + and MEN1−/−) were seeded in a 96-well plate. After 24 h, staurosporine was added to the cells at the final concentration of 1 and 10 µM. The measurement was acquired by FLUOstar OPTIMA (BMG LABTECH, Ortenberg Germany) plate reader for up to 72 h. The date were analyzed by Excel 2016 (Microsoft).

### Statistical analysis

T-test was calculated in order to determine the statistical significance of the results. p < 0.05 value was regarded as significant. The data analysis was performed by Excel 2016 (Microsoft, Redmond, WA USA).

## Supplementary Information

Below is the link to the electronic supplementary material.

Supplementary file1 (TIF 57 kb)

Supplementary file2 (DOCX 11 kb)
