# Supplementary figures and images for: Exploring the MEN1 dependent modulation of caspase 8 and caspase 3 in human pancreatic and murine embryo fibroblast cells
Source: Apoptosis. 2021 Dec 8;27(1-2):70–9. doi: 10.1007/s10495-021-01700-1 (PMC8863690; doi:10.1007/s10495-021-01700-1)

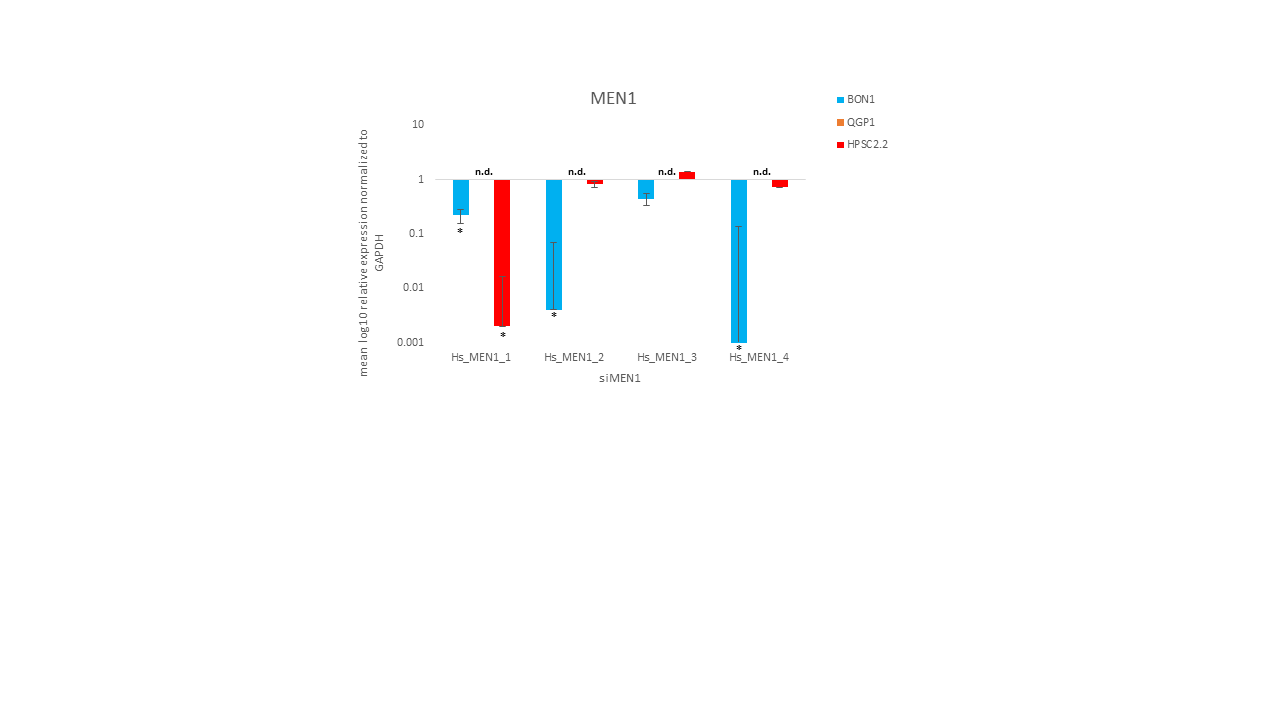

Supplement: Supplementary file 1 — Supplementary file1 (TIF 57 kb) [file 10495_2021_1700_MOESM1_ESM.tif]
