# Supplementary material for: Exploring the MEN1 dependent modulation of caspase 8 and caspase 3 in human pancreatic and murine embryo fibroblast cells
Source: Apoptosis. 2021 Dec 8;27(1-2):70–9. doi: 10.1007/s10495-021-01700-1 (PMC8863690; doi:10.1007/s10495-021-01700-1)
Supplement: Supplementary file 2 — Supplementary file2 (DOCX 11 kb) [file 10495_2021_1700_MOESM2_ESM.docx]

Supplementary Figure Legend

**Supplementary Figure. MEN1 knockdown.**

BON1, QGP1 and HPSC2.2 cells were transfected with four different specific siRNAs for *MEN1* by following the fast transfection protocol of the manufacturer (Qiagen). Shown are means ± SEM of three independent experiments performed with biological duplicates. *p < 0.05 NTC (negative transfection control) vs siMEN1. N.d.: not detectable.
